# Recombinant antigen-based lateral flow tests for the detection of *Strongyloides stercoralis* infection

**DOI:** 10.1371/journal.pntd.0013018

**Published:** 2025-04-08

**Authors:** Robertine Lontuo-Fogang, Thomas B. Nutman

**Affiliations:** Laboratory of Parasitic Diseases, NIAID, National Institutes of Health, Bethesda, Maryland, United States of America; George Washington University School of Medicine and Health Sciences, UNITED STATES OF AMERICA

## Abstract

**Background:**

Strongyloidiasis is a serious public health issue affecting millions of people worldwide particularly in tropical and subtropical regions. The laboratory diagnosis of strongyloidiasis is often serologically based, typically by enzyme linked immunosorbent assays (ELISA). However, the use of these assays at the point of care requires significantly different approaches for serologic measurements. We sought to determine the diagnostic performance of 2 prototype lateral flow tests alongside the Strongy Detect ELISAs (IgG and IgG4) that uses a cocktail of 2 Ss-specific recombinant antigens, Ss-NIE and Ss-IR.

**Methods:**

The diagnostic performance of the Rapid Diagnostic Tests (RDTs) and ELISAs was determined by using stored serum samples from 17 healthy volunteers, 77 individuals known to be stool positive for *Strongyloides stercoralis* (Ss)*,* 44 Ss stool-negative individuals but positive for *Loa loa* (n=32*),* or other helminths (n=12) (hookworm infection, *Schistosoma mansoni*, or *Wuchereria bancrofti)*. Concordance between the RDTs and ELISAs was calculated with the Cohen’s kappa statistic (κ).

**Results:**

The sensitivity and specificity of the IgG RDT was 95% (95% CI; 87.84 to 98.64%) and 94% (95% CI; 84.99 to 98.30%) respectively. The IgG4 RDT showed a sensitivity of 86.5% (95% CI; 77.63 to 92.83%) with 100% (95% CI; 94.13 to 100%) specificity. The IgG-based ELISA showed 100% (95% CI; 95.6-100%) sensitivity and 96% specificity (95% CI; 91.7-98%), whereas the IgG4-based ELISA revealed a 90% (95% CI; 81-94.3%) sensitivity with 100% (95% CI; 97.8-100%) specificity. Concordance between the RDTs and the ELISAs was excellent with κ = 0.94 (95% CI; 0.88-1.0%) for the IgGs and κ = 0.89 (95% CI; 0.81-0.97%) for IgG4 assays.

**Conclusion:**

Given the high degree of sensitivity and specificity of both the IgG- and IgG4-based RDT, either of these would be useful in assessing Ss seropositivity in population-based studies and in screening patients at the point of contact.

## Summary

*Strongyloides stercoralis* (Ss) is a nematode parasite threatening the lives of millions of people in the tropical and subtropical regions of the world. Because of its autoinfective potential, this parasite can live in its host for many years, producing larvae that mature into adult worms within the host without the need for reinfection. Diagnosis of this parasite is challenging, relying on the detection of larvae in stool. Alternatively, serology has been very useful through the detection of antibodies produced against this parasite. The present study evaluates the diagnostic performance of 2 new lateral flow tests (Strongy Detect Rapid Tests) in comparison with the now commercially available Strongy Detect ELISAs. We used stored serum samples from Ss positive individuals, healthy controls and subjects infected with other helminth parasites. These lateral flow assays were very accurate at identifying Ss-specific antibodies in samples known to have the infection. There was an excellent agreement between these new lateral flow tests and the commercially available ELISAs. The excellent performance of these new tests makes them good diagnostic tools for use at point of care and in laboratory settings.

## Introduction

Strongyloidiasis, caused by the parasite *Strongyloides stercoralis* (Ss), is a neglected tropical disease (NTD) with an estimated 613.9 million people infected worldwide [1]. Most of these infections occur in low resource areas in Africa, Latin America and Asia, even though most of these countries still have missing information on its epidemiology due to the lack of appropriate diagnostic tools [1–3]. This has led to underestimation of the disease burden driving an urgent need for fast and affordable tests to allow for improved estimates of disease around the world. Infection with Ss is commonly subclinical but can become life threatening in immunocompromised individuals due to its propensity for autoinfection [4,5]. The parasitologic diagnosis of Ss is challenging and require sophisticated methods (qPCR) and time-intensive methodologies such as the Baermann concentration or agar plate culture [6].

The development of tools that can be used for diagnosis at point of care and assessing Ss public health burden, especially in low resource settings, is essential. Recently there has been growing interest in the development of new immune-based diagnostics to improve the detection of strongyloidiasis. Enzyme Linked Immunosorbent Assays (ELISAs) tests have been the mainstay of the diagnostic armamentarium, although the need for multiplate readers, the cold chain for reagents, a centralized laboratory facility and trained personnel often offsets the benefits of the improved diagnostic capabilities these ELISAs offer [7–9]. Several lateral flow tests have been developed for the diagnosis of strongyloidiasis [10,11] that measure IgG [12–14] and IgG4 antibodies [7,12,15] to Ss-specific recombinant antigens (e.g., Ss-NIE and/or Ss-IR [16–18], most of which are still not commercially available. Previously, a laboratory-based assessment of two prototype recombinant Ss-NIE- and Ss-IR- based ELISAs for the detection of Ss (Strongy Detect IgG and IgG4 ELISAs) were validated showing a diagnostic sensitivity and specificity of 99% for the IgG-based assay and a sensitivity of 96% with an 100% specificity for the IgG4 immunoassay [9]. These ELISAs, have shown to be quite promising for use in the field with assays being reproducible with no systematic border effect in a technical evaluation study [19]. These ELISAs are coated with both Ss-NIE and Ss-IR recombinant antigens for the detection of either IgG or IgG4 antibodies. In the present study, we demonstrated the diagnostic performance of prototype recombinant antigen-based Strongy Detect RDTs alongside the now commercially available Strongy Detect IgG and IgG4 ELISAs.

## Methods

### Ethics statement

Serum samples were collected as part of several National Institutes of Health (NIH) Intramural IRB-approved studies performed under the auspices of the Laboratory of Parasitic Diseases, National Institute of Allergy and Infectious Diseases (NAID) (NCT00001645, NCT00001230). All patients or parent/guardian of patients provided informed written consent.

### Serum samples

Based on sample availability and the number of RDTs donated, a total of 138 sera sets were tested on both the prototype Strongy Detect Rapid tests and the ELISAs (all donated by InBios International). For this, we used stored serum samples collected from 77 Ss stool positive individuals (true positives) and 61 Ss negative samples but positive for other helminths (true negatives). The true negatives were made of 17 healthy volunteers who were free from any intestinal helminth infections at the time of blood collection, and 44 subjects that had parasitologically proven infection with *Loa loa* (n=32), or single infection with other helminths (n=12) including hookworms, *Schistosoma* spp. or *W. bancrofti.* (Table 1 and S1-S3 Figs). In addition to this, 126 more samples were tested solely on the Strongy Detect ELISAs, totaling 264 samples for the ELISAs (S1 Table).

**Table 1:** Distribution of serum samples among the different groups involved in the evaluation of Strongy Detect ELISAs and Rapid tests.

Parasite/type	Number tested
*Strongyloides stercoralis*	77
*Loa loa*	32
*Wuchereria bancrofti*	5
*Schistosoma mansoni*	1
Hookworms	6
Healthy controls	17
**Total**	**138**

### Rapid diagnostic tests

**Fig 1:**
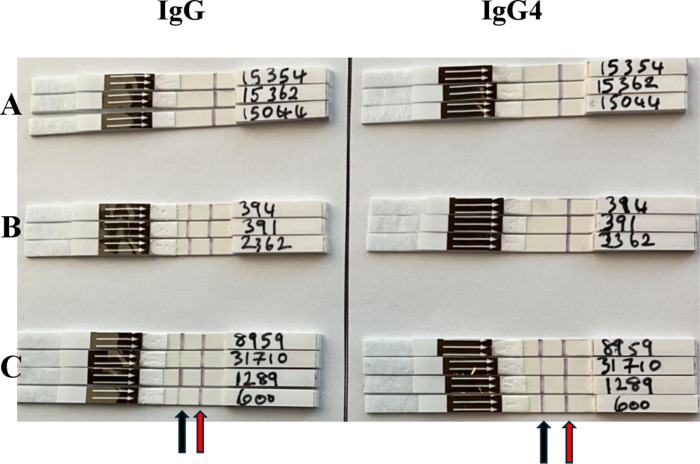
Appearance of representative Strongy Detect IgG (left panel) and IgG4 (right panel) Rapid dipsticks following application of serum samples. Tests showing negative results from uninfected controls for both IgG and IgG4 (A, n=3). Tests showing positivity for IgG but not IgG4 (B, n=3) and Dipsticks showing positivity in both IgG and IgG4 strips (C, n=4). Red arrows indicate the control lines and black arrows are the test lines (Credit: Robertine Lontuo-Fogang).

Strongy Detect IgG and IgG4 Rapid tests are membrane-based immunoassays coated with SsNIE and SsIR recombinant antigens for the qualitative detection of *Strongyloides stercoralis*-specific IgG and IgG4 antibodies in whole blood and serum. During the test, 15 μL of serum was applied to the sample pad with the strip kept in a horizontal position, making sure all the serum was absorbed. Three drops of a “Chase Buffer” were added to a microtiter well and the test strip placed in the well in a vertical position. Strips were allowed to stand in this position undisturbed and the results were read in 15–20 mins. The test was considered positive if both the control and test lines appeared (Fig 1).

### ELISA immunoassays

The IgG and IgG4 Strongy Detect ELISA kits coated with Ss-NIE and Ss-IR recombinant antigens were conducted as indicated by InBios International Inc. Samples and controls were run in duplicate by diluting them at 1/25 for IgG4 and 1/100 for IgG in the ELISA sample diluent provided in the kit. One hundred microliters of diluted sera, negative controls and positive controls were added on to the plate in duplicate. Each plate had 4 blank wells onto which only the sample diluent was added. The plate was covered and incubated at 37^o^C for 30 mins. Plates were then washed 6 times using 1X wash buffer, followed by the addition of 100 μL anti-human IgG or IgG4 antibodies conjugated to Horse Radish Peroxidase (HRP) and incubated at 37^o^C for another 30 mins. Plates were again washed, 100 μL of liquid Tetramethylbenzidine (TMB) added and incubated at room temperature in the dark for 10 mins. After incubation, 50 μL of 1N sulfuric acid was added and incubated at room temperature for 1 min to stop the reaction. Raw optical densities (ODs) were read at 450 nm with SpectraMax i3 spectrometer (Molecular Devices, California). Signal to noise ratio (S/N) were calculated by dividing the mean ODs of the samples by the mean ODs of the plate blanks. Cutoff values were determined using the Youden’s J index.

### Statistical analysis

Youden’s J cutoffs were obtained for the ELISA immunoassays using the Epitools diagnostic test evaluation and comparison tools (https://epitools.ausvet.com.au/roccurves). The Receiver Operator Curves (ROC) were generated in Prism version 10.4.1 and the Wilson/Brown method [20] was used to calculate the confidence intervals. Geometric means were used for central tendency measures. All statistical analyses were performed with Prism version 10.4.1 with the two-sided p<0.05 level of significance. Concordance between the ELISAs and the RDTs was assessed using the Cohen’s kappa statistic and the results interpreted as described hereafter: < 0 = less than chance agreement, 0.01-0.2 = slight agreement, 0.21-0.4 = fair agreement, 0.41-0.6 = moderate agreement, 0.61-.0.8 = substantial agreement and 0.81-0.99 = almost perfect agreement.

## Results

### Strongy Detect IgG and IgG4 Rapid tests

When the 138 serum samples were run on the prototype Strongy Detect IgG and IgG4 Rapid tests, the IgG Rapid test showed a sensitivity of 95% (95% CI; 87.84-98.64%) and specificity of 94% (95% CI; 84.99-98.30%) in the detection of *S. stercoralis*-specific IgG antibodies. The Strongy Detect IgG4 Rapid tests in contrast showed a sensitivity of 86.5% (95% CI; 77.63-92.83%) and a specificity of 100% (95% CI; 94.13-100%).

### Strongy Detect IgG and IgG4 ELISA assays

The prototype ELISAs were previously validated in our lab revealing sensitivity of 99% and 96% for IgG and IgG4 respectively [9]. Here, 264 stored serum samples were used to establish cutoffs for positivity and negativity for the revised version of the Strongy Detect ELISAs. The diagnostic sensitivity and specificity of these immunoassays were analyzed from raw Optical Densities (ODs) and Signal/Noise ratio (S/N) using the blanks obtained from each plate. In this study, we use the Youden J cutoffs for both S/N and ODs obtained from the Epitool online sensitivity and specificity calculator. The cutoffs were 0.37 for IgG and 0.44 for IgG4 ODs and 8.5 and 11.1 from the SN respectively for the IgG and IgG4 ELISAs. Based on these cutoffs and regardless of the parameter (OD or S/N) used, the Strongy Detect IgG ELISA showed a sensitivity of 100% (95% CI 95.6-100%) and a specificity of 96% (95% CI 91.7-98%) and the Strongy Detect IgG4 ELISA showed a sensitivity of 90% (95% CI 81-94.3%) and a specificity of 100% (95% CI 97.8-100%).

**Fig 2:**
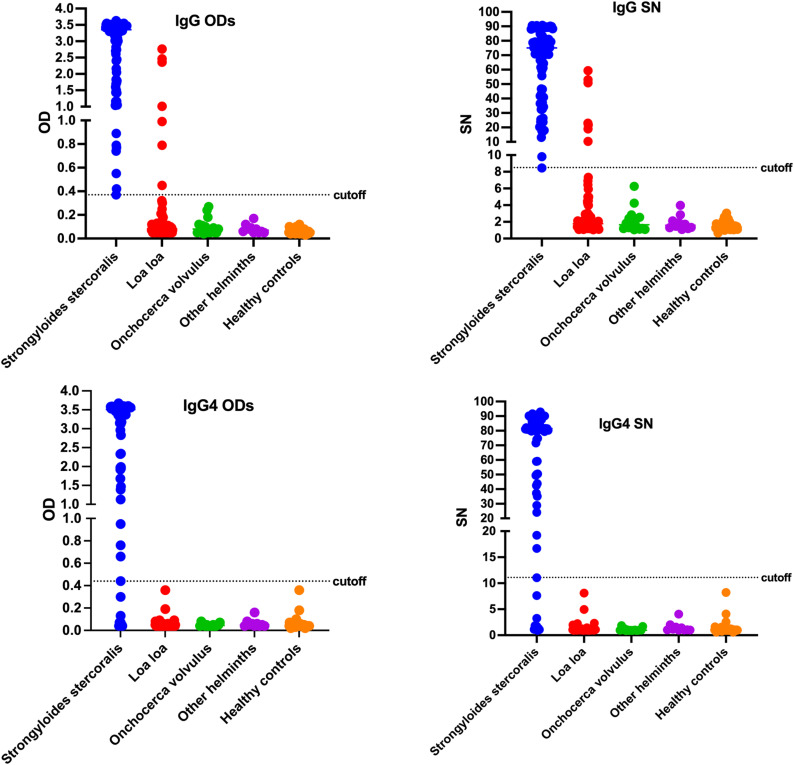
Performance of second generation Strongy Detect IgG and IgG4 ELISAs using Optical Densities (ODs - left panels) or Signal to Noise (SN- right panels) The dashed lines represent Youden J cutoffs of the different tests.

The diagnostic performance of the Strongy Detect ELISA assays is presented on Fig 2. As can be seen, these assays were able to discriminate between Ss positive samples and Ss negative samples quite easily. Nonetheless, in the IgG-based ELISA, 7 *Loa loa*-infected samples were above the cutoff suggesting some serologic cross reactivity with this parasite. Interestingly, no cross reactors were seen in the IgG4-based assays but the false negatives in the Ss positives resulted in the reduced sensitivity. All healthy controls tested negative both in the IgG and IgG4 ELISAs.

**Fig 3:**
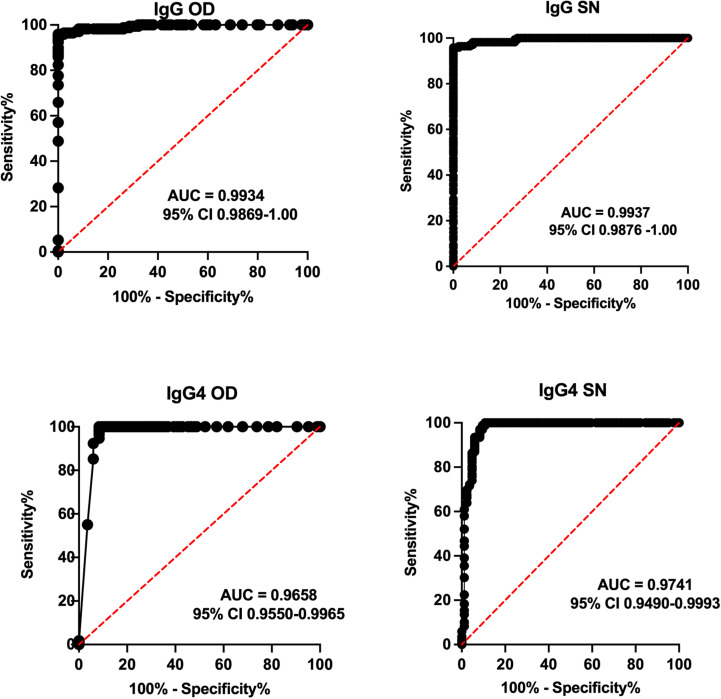
Receiver Operator Curves (ROC) using optical densities (ODs-left panels) and signal to noise (S/N) of Ss positive and Ss negative samples. Area Under Curve (AUC) and confidence intervals (CI) are shown on each graph.

Receiver Operator Curves (ROC) obtained from ODs and S/N of Ss positive individuals against all Ss negatives (both healthy volunteers and those with other helminths) are shown in Fig 3. As can be seen, the Area Under the Curve (AUC) using either ODs or S/N approached 1 (95% CI: 0.98-1; P<0.0001) for the IgG-based ELISA and was 0.97 (95% CI: 95–1; p<0.0001) for the IgG4-based ELISA.

### Concordance between Strongy Detect ELISAs and Rapid tests

An “almost perfect agreement” was reported between the RDTs and the ELISAs with a kappa value of κ = 0.94 (95% CI; 0.88-1.0%) for the IgGs (IgG RDT and IgG ELISA) and κ = 0.89 (95% CI; 0.81-0.97%) for IgG4 assays (IgG4 RDT and IgG4 ELISA).

## Discussion

The diagnosis of strongyloidiasis has been mainly serology-based due to challenges in stool detection methods. Many studies have highlighted the importance of the recombinant Ss-specific antigens (NIE, Ss-IR) in ELISAs given their good diagnostic sensitivities and specificities with reduction in cross reactivity with other helminths (particularly *Loa loa*) [17,21,22]. The present study reports the diagnostic performance of 2 prototype lateral flow tests in comparison with the InBios’ Strongy Detect IgG and IgG4 ELISAs. The Strongy Detect IgG and IgG4 ELISAs are recombinant antigen based immunoassays combining both the NIE and Ss-IR recombinant antigens [9]. Herein, we corroborate the performance of the Inbios’ Strongy Detect ELISAs irrespective of the criteria used (ODs or S/N). These assays showed a sensitivity of 100% and specificity of 96% for the IgG ELISA; for the IgG4 ELISA, the sensitivity dropped to 90% but the specificity reached 100%. This high diagnostic performance can be attributed to the act of combining both the NIE and Ss-IR antigens within a single well of the ELISA plate in these assays. However, the combination of both SsIR and SsNIE recombinant antigens for the detection of Ss total serum IgG have been previously reported but with sensitivity and specificity of 95.0% and 90.2% respectively [18] which is lower compared to the 100% and 96% obtained in this study.

These sensitivities and specificities compare quite favorably to 3 commercially available IgG ELISA kits using crude extracts of *Strongyloides* spp parasites, whereby, in a recent study by Weitzel et al. [23], sensitivities in these assays ranged from 56% to 64% and specificities from 92% to 96% [23].

Just like many other studies [17,24–26], we reported a higher sensitivity for the IgG ELISA and a higher specificity for IgG4 in this study. The use of IgG4-based assays to improve specificities in chronic helminth infection have been reported in a host of studies including those for Ss [25], *Loa loa* [26], *Onchocerca volvulus*[27] and *Wuchereria bancrofti* [28]. IgG4 ELISAs have not only proven to increase specificity but also have shown reduced cross-reactivity with other helminths [11,23]. Despite the high sensitivity of the IgG ELISA, it revealed some cross reactivity in 7 of the samples infected with *Loa loa,* which is not uncommon as reported in previous studies [17,25]. In fact, Weitzel et al [23] reported cross reactions of up to 42.5% in nematodes, 38.1% in trematodes and 32.5% in cestodes in 3 commercially available IgG ELISAs. Interestingly, no cross reactivity was reported in the IgG4 ELISA. Another remarkable point in this study is the significant area under the curve of ~1 in both IgG and IgG4 ELISAs implying an excellent diagnostic accuracy of these assays.

Although ELISAs can be very useful for the detection and quantification of antibodies in patient samples, there is a need for simple and field applicable tools that can be easily applied at point of care. ELISAs can be time consuming and not suitable for individual screening. While several prototype point of care tests for the detection *Strongyloides stercoralis* have been reported [7,12,13], these are still not commercially available [29]. In the present study, a preliminary assessment of 2 prototype lateral flow tests (Strongy Detect IgG and IgG4 Rapid tests) was performed and shown to have a diagnostic sensitivity of 95% and a 94% specificity for the Strongy Detect IgG Rapid test. For the Strongy Detect IgG4 Rapid test, there was a 100% specificity but only an 86.5% sensitivity. These parameters show a significant improvement that has been reported previously by others [1,12,13] in IgG Immunochromatographic Rapid tests. Indeed our results are consistent with the findings of Scarso *et al* [30] reporting the accuracy of these RDTs with a higher sensitivity (91.1%) for the IgG compared to the Ig4 (77.3%) and a 100% specificity (95% CI; 92.1-100%) for IgG4 RDT suggesting its use in prevalence studies in endemic zones.

An IgG4-based lateral flow test (Ss Rapid) that makes use of a combination of rNIE and another less well-defined antigen (rSs-1a) reported sensitivities ≥82% and specificities ≥94% [7,15,29,31]. These findings parallel our own findings using the Strongy Detect IgG4 Rapid test. Other results that are worth noting in the study are the high concordance between the IgG and IgG4 immunoassays translating their accuracy and consistency in detecting true positives and true negatives. Perhaps, this agreement between the RDTs and ELISAs is demonstrated consistently through the higher sensitivities when detecting IgG and higher specificities when detecting IgG4. However, the use of each RDT might fit in different situations. High sensitivity tests might be recommended for individual screening of patients about to undergo surgery/organ transplant so as to minimize the risk of undiagnosed infections [32] and also avoid the spread of infection from the donor to the recipient [33] as would studies on prevalence in endemic areas for high specificity tests [34].

Taken together, the excellent performance of the Strongy Detect Rapid tests make them suitable diagnostic tools at both the point of care or in laboratory settings. These prototypes are now being built into a more robust cassette form to allow for POC assessment/validation and to minimize complexity during field application.

## Conclusion

The present study shows an initial validation of 2 prototype Strongy Detect Rapid tests in comparison with Strongy Detect ELISAs with very high sensitivities and specificities in the detection of *Strongyloides stercoralis* specific antibodies. Given that the detection of Ss-specific IgG is more sensitive but less specific and that of IgG4 is more specific, the combination of both immunoassays will be useful for an improved serodiagnosis for patient screening and epidemiological studies in the less developed countries harboring most of the disease burden.

## Supporting information

S1 Table Distribution of the 126 extra serum samples tested exclusively on the Strongy Detect ELISAs. (DOCX)

S1 Fig Image of Strongy Detect Rapid Dipsticks showing all 77 true positives (*Strongyloides stercoralis* positive) after application of serum samples (Credit: Robertine Lontuo-Fogang). (TIFF)

S2 Fig Appearance of Rapid dipsticks showing all 17 healthy controls (true negatives) following application of serum (Credit: Robertine Lontuo-Fogang). (TIFF)

S3 Fig Appearance of Rapid dipsticks tested on all 44 *Strongyloides stercoralis* negative (true negatives) samples but infected with other helminths (Credit: Robertine Lontuo-Fogang). (TIFF)

S1 Data Minimal data sets for ELISAs. (XLSX)
