# Supplementary material for: Recombinant antigen-based lateral flow tests for the detection of Strongyloides stercoralis infection
Source: PLoS Negl Trop Dis. 2025 Apr 8;19(4):e0013018. doi: 10.1371/journal.pntd.0013018 (PMC12011289; doi:10.1371/journal.pntd.0013018)
Supplement: S1 Table — (DOCX) [file pntd.0013018.s001.docx]

Supplementary Table 1: Distribution of the 126 extra serum samples tested exclusively on the Strongy Detect ELISAs

| **Parasite/type** | **Number tested** |
| --- | --- |
| *Strongyloides stercoralis* | 7 |
| *Onchocerca volvulus* | 16 |
| *Loa loa* | 58 |
| *Taenia solium* | 3 |
| *Wuchereria bancrofti* | 5 |
| *Schistosoma mansoni* | 0 |
| Hookworms | 0 |
| Healthy controls | 37 |
| **Total** | **126** |
