# Supplementary figures and images for: Recombinant antigen-based lateral flow tests for the detection of Strongyloides stercoralis infection
Source: PLoS Negl Trop Dis. 2025 Apr 8;19(4):e0013018. doi: 10.1371/journal.pntd.0013018 (PMC12011289; doi:10.1371/journal.pntd.0013018)

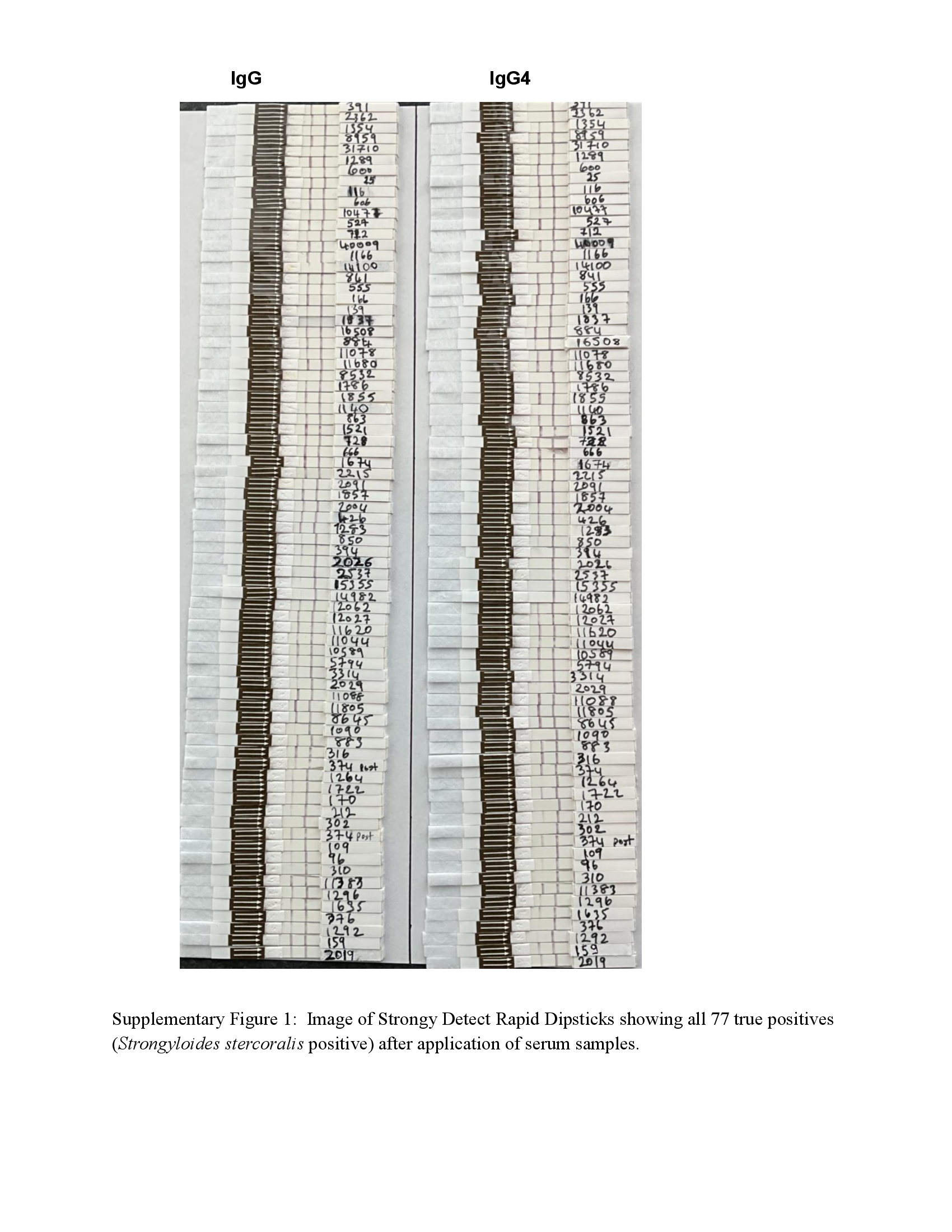

Supplement: S1 Fig — (TIFF) [file pntd.0013018.s002.tiff]

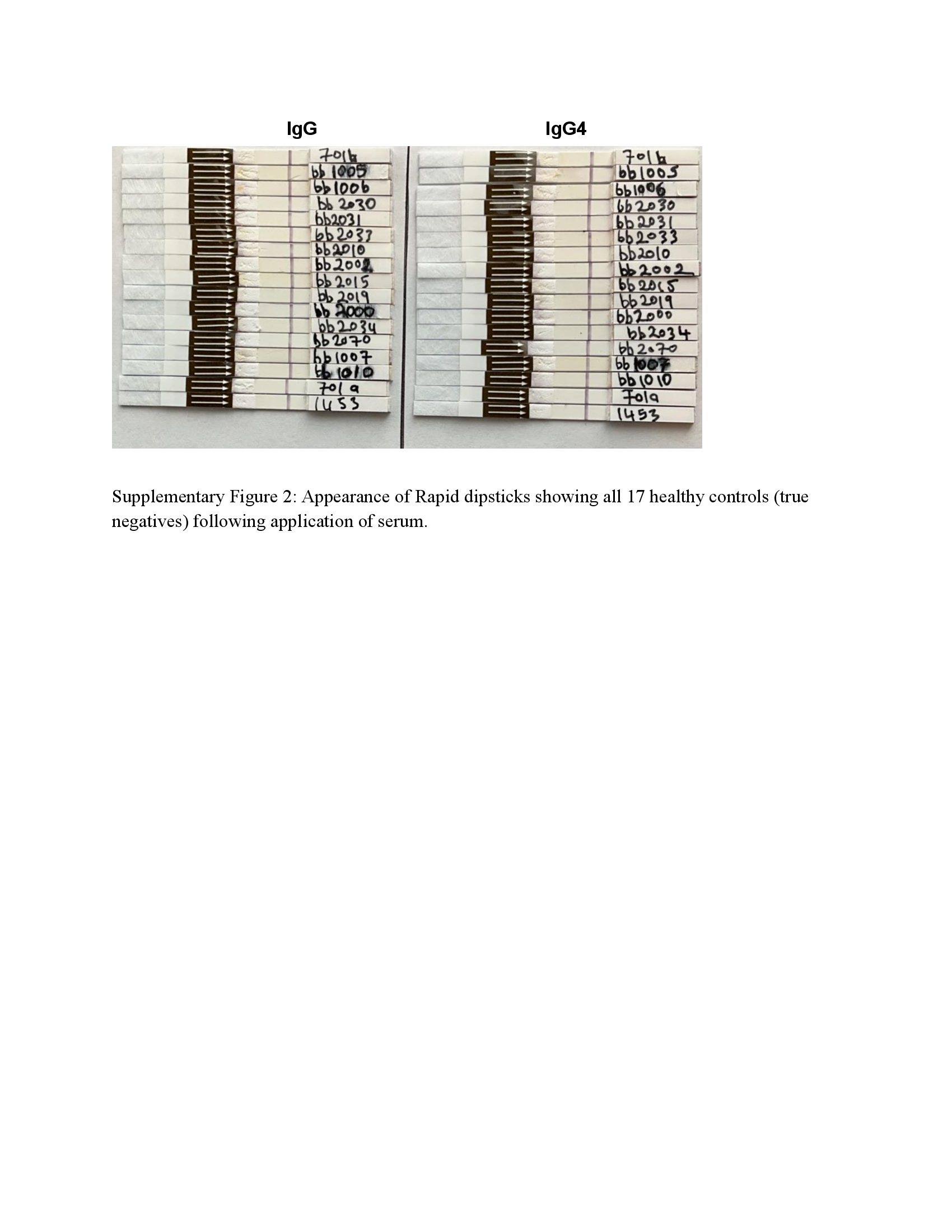

Supplement: S2 Fig — (TIFF) [file pntd.0013018.s003.tiff]

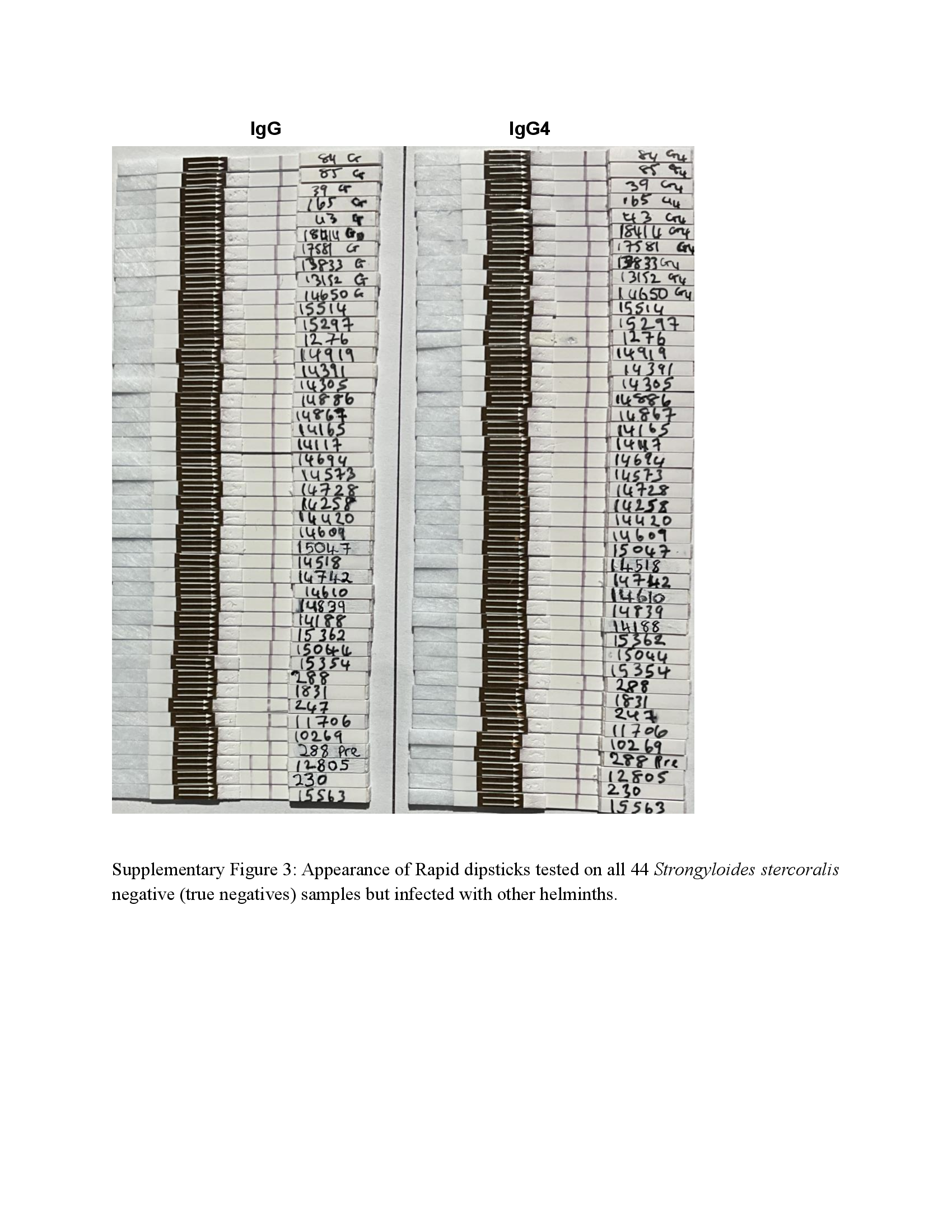

Supplement: S3 Fig — (TIFF) [file pntd.0013018.s004.tiff]
